# Linear and Non-Linear Heart Rate Variability Indexes from Heart-Induced Mechanical Signals Recorded with a Skin-Interfaced IMU †

**DOI:** 10.3390/s23031615

**Published:** 2023-02-02

**Authors:** Čukić Milena, Chiara Romano, Francesca De Tommasi, Massimiliano Carassiti, Domenico Formica, Emiliano Schena, Carlo Massaroni

**Affiliations:** 1 Empa Materials Science and Technology, Biomimetic Membranes and Textiles, 9014 St. Gallen, Switzerland; 2 3EGA B.V., 1062 KS Amsterdam, The Netherlands; 3 Unit of Measurements and Biomedical Instrumentation, Università Campus Bio-Medico di Roma, Via Alvaro del Portillo, 00128 Rome, Italy; 4 Unit of Anesthesia, Intensive Care and Pain Management, Università Campus Bio-Medico di Roma, Via Alvaro del Portillo, 00128 Rome, Italy; 5 School of Engineering, Newcastle University, Newcastle upon Tyne NE1 7RU, UK

**Keywords:** heart rate, heart rate variability, seismocardiography, gyrocardiography, inertial units, HRV, linear HRV, non-linear HRV

## Abstract

Heart rate variability (HRV) indexes are becoming useful in various applications, from better diagnosis and prevention of diseases to predicting stress levels. Typically, HRV indexes are retrieved from the heart’s electrical activity collected with an electrocardiographic signal (ECG). Heart-induced mechanical signals recorded from the body’s surface can be utilized to record the mechanical activity of the heart and, in turn, extract HRV indexes from interbeat intervals (IBIs). Among others, accelerometers and gyroscopes can be used to register IBIs from precordial accelerations and chest wall angular velocities. However, unlike electrical signals, the morphology of mechanical ones is strongly affected by body posture. In this paper, we investigated the feasibility of estimating the most common linear and non-linear HRV indexes from accelerometer and gyroscope data collected with a wearable skin-interfaced Inertial Measurement Unit (IMU) positioned at the xiphoid level. Data were collected from 21 healthy volunteers assuming two common postures (i.e., seated and lying). Results show that using the gyroscope signal in the lying posture allows accurate results in estimating IBIs, thus allowing extracting of linear and non-linear HRV parameters that are not statistically significantly different from those extracted from reference ECG.

## 1. Introduction

Cardiovascular diseases are confirmed to be one of the leading causes of mortality worldwide, resulting in more than 17 million deaths globally [1,2]. It has been known for quite a long time that behind cardiovascular mortality and dysfunction are aberrated dynamics of the autonomic nervous system (ANS) [3,4]. The tenth cranial nerve, the vagus nerve, has a crucial impact on heart and breathing dynamics (that are also coupled and synchronized in a healthy person) among other internal systems of organs that are innervated with its several branches, both above and below the diaphragm (myelinated and unmyelinated branches of the vagus nerve). Cortico-vagal control of heart dynamics via vagus nerve exercise has both stimulatory and inhibitory effects on cardiac tissue (i.e., sympathetic and parasympathetic system activity), driving an increase or decrease in heart rate (HR) [5].

The monitoring of HR and its variability (HRV) proved to be a prognostic as well as preventative factor in several cardiovascular diseases with important implications from healthcare to sport [6,7]. From the variabilities of time intervals between the two consecutive heartbeats (inter-beat intervals, IBI, or RR intervals), HRV indexes can be estimated. HRV has been found to be able to capture fast fluctuations that may be an indication of sympathetic and vagal activity and, in turn, to assess cardiac abnormalities with established applicability, among others, in risk stratification of cardiovascular patients [8,9], monitoring of depression [10,11,12], and for diagnosis and monitoring of performance in sport and physical activities [13].

Traditional HRV measures are usually divided into two categories: linear and non-linear [14]. HRV can be evaluated in the time or frequency domain using linear techniques. Time domain indexes are statistical calculations of consecutive IBIs values that represent the simplest way to calculate HRV, whereas frequency domain indexes are more extensive indexes based on spectral analysis that is generally used to assess the contribution of the autonomic nervous system to HRV. Non-linear indexes, on the other hand, are based on mathematical approaches that are not impacted by nonstationarity, as is the case with linear indexes. Non-linear indexes, as opposed to linear indexes, take into account minor fluctuations that represent the fundamental features of cardiac dynamics caused by continual competition between opposite autonomous nervous system components (parasympathetic and sympathetic drive). Additional details on the importance and significance of the HRV parameters can be found in [14].

From the sensor side, generally, ECG waveforms that represent the heart’s electrical activity are processed to extract IBIs first and then calculate HRV indexes [15]. However, the recent advances in sensors and miniaturized electronics have encouraged the investigation of new approaches and measuring techniques to be applied in the cardiology field for monitoring HR and IBIs leveraging both contact-based and contactless techniques. Conversely to ECG, which reflects the electrical activity of the heart, other techniques measuring (directly or indirectly) the heart-induced mechanical signals can be used [16,17,18,19,20]. Among others, accelerometers and gyroscopes in the last decade have been explored to extract information about the mechanical phases of the cardiac cycle from precordial vibrations captured at the chest level and changes in its angular velocities, respectively. These signals are generally known as seismocardiogram (in short SCG) [21,22,23] and gyrocardiogram (in short GCG) [24], respectively. For instance, when rightly collected from both the SCG and GCG signals, it is possible to identify some key cardiac events during both the systole and diastole [22,24,25]. In the systolic phase of the cardiac cycle, SCG and GCG may allow the identification of the mitral valve closure (MC), the isovolumic moment (IM), the aortic valve opening (AO), the isotonic contraction (IC), and the rapid ejection (RE) [22,26].

GCG and SCG are not yet standard in cardiology, but they are promising since they can augment monitoring and more accurate insights in both respiration and heart dynamics much needed in cardiology and particularly in every ICT Unit. An Inertial Measurement Unit (IMU) generally embeds both an accelerometer and gyroscope and is a perfect candidate to record both the SCG and GCG and then extract not only HR values but also HRV indexes. Not surprisingly, in the last decade, few studies started investigating the feasibility of providing accurate IBI and HRV values also through SCG and GCG signals, mainly from data provided by databases (e.g., CEBS) and standard posture [27,28,29,30,31]. Only a few studies compared the HRV indexes extracted from ECG and contextually from SCG and GCG but with cumbersome sensors and/or without providing an exhaustive comparison for non-linear indexes [26,32,33]. In a previous paper, we tried to estimate some linear HRV indexes from both the SCG and GCG in a very small cohort of volunteers [34]. Literature evidence lacks some in terms of the absence of studies on fractal analysis and several entropy-based measures relying on non-linear HRV indexes extracted from SCG and GCG, the absence of studies investigating the influence of common posture assumed by patients/users (i.e., seated and supine) on both SCG and GCG contextually used for HRV index extraction [17]. As per the last reason, although it is known that the waveforms of SCG and GCG are strongly influenced by the posture assumed by the subject [24,33,35], the majority of studies neglected possible sources of inaccuracies deriving from the wrong selection of accelerometer and gyroscope axis for HRV estimation. All these limitations are still hindering the possible use of IMU for extraction of IBI and HRV values in possible real-world scenarios as an alternative to ECG.

This paper aims at investigating the feasibility of estimating the most common linear and non-linear HRV indexes from SCG and GCG signals acquired from healthy volunteers during two resting postures (i.e., seated and lying). Since the extraction of HRV indexes relies on the estimation of IBIs from the raw SCG and GCG signals and the morphology of these signals depends on the body posture, we investigated the performances of two different envelope-based algorithms for IBIs extraction (Algorithm 1 using standard axis for SCG and GCG extraction, Algorithm 2 automatically identifying the best axis for the SCG and GCG computation) to identify the most promising signal and posture to extract accurate HRV parameters when compared to reference ECG data.

## 2. Materials and Methods

Twenty-one healthy volunteers (18 males and 3 females, mean age: 27 ± 5 years, mean height: 177 ± 9 cm, mean body mass: 74 ± 15 kg, mean BMI: 23 ± 3 kg/m^2^) were enrolled in this study. Each volunteer underwent two different trials, assuming two common postures while performing vital signs monitoring at home and in the clinical environment: (1) seated posture and (2) lying posture.

A single IMU (Xsens DOT by Xsens, The Netherlands) was attached with a hypoallergenic medical-grade double-sided tape at the xiphoid process level. Among the numerous body landmarks used to collect cardiac-related acceleration signals, we used the xiphoid because of good inter-subject reliability and easy identification of the body [21,22]. The selected IMU sensor has different features that make it ideal in this scenario. This IMU embeds a tri-axis accelerometer (full scale ±16 g) and a tri-axis gyroscope (full scale ±2000 °/s) with dimensions (36 × 30 × 11 mm) and mass (11.2 g) perfect for wearing. The IMU sensor was programmed via Bluetooth Low Energy 5.0 to store raw accelerations and angular velocity data inside the onboard memory with a sampling rate of 120 Hz [36]. At the same time, a reference wearable device (Bioharness v3 by Medtronic, Minneapolis, MN, USA) was worn around the chest to record the reference ECG signal at a 250 Hz sampling rate and the acceleration at the level of the chest at 100 Hz inside the onboard memory. Bioharness uses two dry electrodes and dedicated electronics to capture one-lead ECG and a triaxial accelerometer (full scale ±16 g) to record the linear acceleration of the trunk [37].

**Figure 1:**
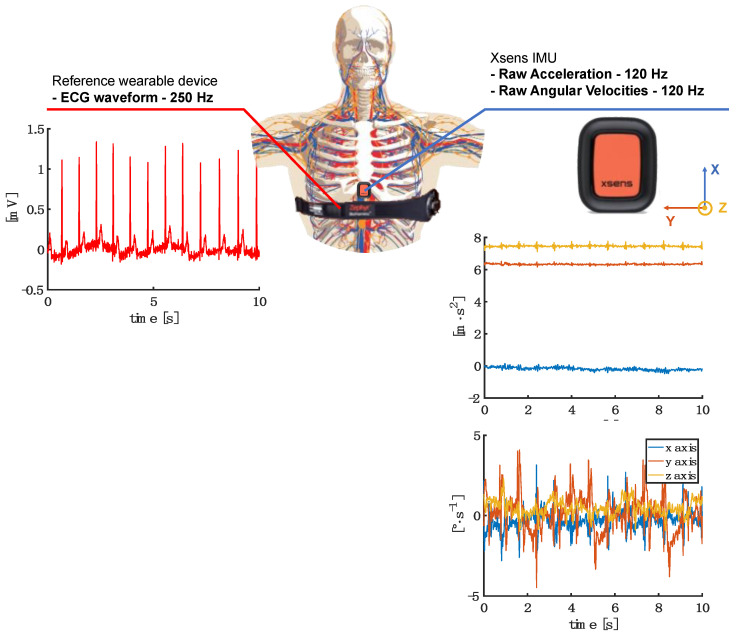
Schematic representation of the positioning of the sensors on the subject’s body and raw waveforms obtained from the ECG sensor sampled at 250 Hz (red waveform on the left) and the IMU sensor for both the accelerometer sensor and the gyroscope sampled at 120 Hz. For accelerometers and gyroscopes, all three axes are shown (x in blue, y in orange, and z in yellow). Regarding the IMU sensor, the *x*-axis is oriented from foot to head, the *y*-axis laterally from left to right, and the *z*-axis is oriented from back to chest.

After wearing the IMU sensor and reference wearable device (see Figure 1), volunteers were left free to wear shirts or other clothing on their torsos to make the measurement as comfortable as possible. Each volunteer underwent two different trials, assuming two common postures in working and clinical environments: (1) seated posture and (2) lying posture. In each trial, volunteers were called to breathe for 120 s after an initial end-inspiratory apnea used to synchronize the reference ECG signal with the IMU signals.

The study was conducted in accordance with the Declaration of Helsinki, and the study design was approved by the Ethical Committee of Università Campus Bio-Medico di Roma (code: 27.2(18).20 of 15 June 2020).

### 2.1. SCG and GCG Signals

In the ECG’s electrical signal, the most prominent peak results from the major ventricular depolarization in the electro bio-potential measurement (R peak). Similarly, AO is the most evident peak in both SCG and GCG signals since it is caused by an acceleration increase happening after the basal depolarization of ventricles, causing build up pressure, inward movement of the apex, and swelling of the walls. One open challenge is related to the particular morphology of both the SCG and GCG [24,35,38]. Different factors, such as cardiovascular diseases, the posture assumed by the subject, and the noise on the signal may strongly affect the prominence of the AO peak. Although several methods have been proposed [39], the accuracy in detecting AO is still low, even applying complicated algorithms when the data are collected in real-world scenarios. What is common is that MC or RE are higher in amplitude when compared to AO. This may cause errors in estimating IBIs up to 100 ms, which makes SCG and GCG unreliable in estimating HRV indexes [40,41,42].

To tackle this open problem, in this paper, we applied two algorithms to first extract SCG from accelerometer data and GCG from gyroscope ones, and then detect AO fiducial points from the signals.

Prior to the data analysis, we verified the presence of involuntary motion artifacts caused by the trunk movements of each volunteer during the data collection in lying and seated postures. At this scope, we calculated the Vector Magnitude Unit (*VMU*) from the *x*-, *y*-, and *z*-axis of the Bioharness accelerometer for estimating the level of activity in accordance with the following equation:

(1) $$VMU=\sqrt{a_{x}^{2}+a_{y}^{2}+a_{z}^{2}}$$

where $a_{x}$, $a_{y}$, $a_{z}\;$are the acceleration components along the *x*-, *y*-, and *z*-axis. To compute the *VMU*, the raw Bioharness accelerometer signals were filtered to remove the gravity components. Only the signals with VMU<0.1 g  were considered as resting activities without motion artifacts [43,44]. In all the trials (42 in total, 2 min each) considered for the analysis we always found VMU<0.05 g. Therefore, we processed all the data from the IMU and the reference ECG without excluding any part of the collected signals for the subsequent analysis.

First, the raw skin-interfaced IMU accelerometer and gyroscope signals were band-pass filtered with an FIR filter with cut-off frequencies between 4 Hz and 30 Hz which contains most frequencies associated with mechanical activity of the heart as widely reported in the literature [35,45].

**Figure 2:**
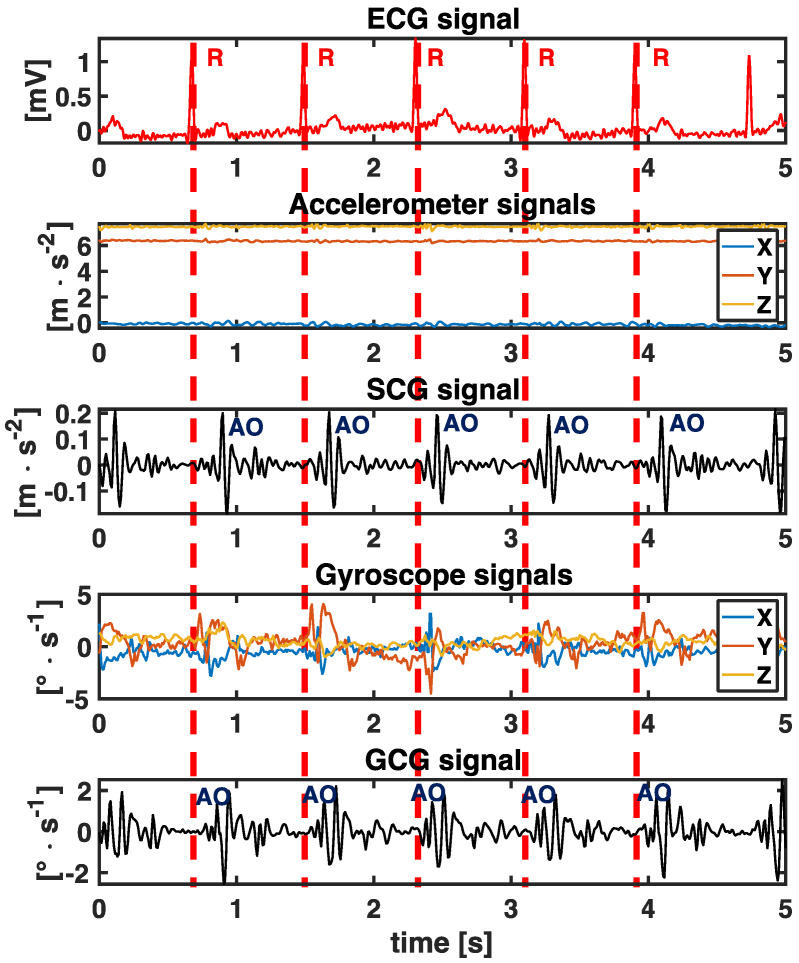
5 s of collected data from a volunteer in a lying position. First subplot: ECG waveform with detected R peaks. The R-R peaks distance allows calculating $IBI_{RR,i}$. Second subplot: raw accelerations along the *x*-, *y*-, and *z*-axes. Third subplot: the obtained SCG signal with detected AO peaks. The AO-AO peaks distance allows calculating $IBI_{AOAO,i}$. Fourth subplot: raw angular velocities along the *x*-, *y*-, and *z*-axes. Fifth subplot: the obtained GCG signal with detected AO peaks. The AO-AO peaks distance allow calculating $IBI_{AOAO,i}$.

Figure 2 reports the raw accelerometer and gyroscope signals, as well as SCG and GCG ones, compared to the ECG signal. The AO and R peaks are highlighted in the first 5 events. It is well known that the AO events are 150–250 ms after the ventricular depolarization events (R peaks) [46]. The accurate identification of AO peaks from SCG and GCG signals is crucial to estimate the time elapsed between two AO events correctly ($t_{AO}{|}_{n}-t_{AO}{|}_{n-1}$) and then obtain reliable HRV measurements. As stated in previous articles, the main issues dealing with SCG and GCG are the morphology of the waveform at each beat since those are influenced by a variety of factors [22,38,47,48].

The two algorithms implemented in this article aim to detect AO peak instants without an R-wave of ECG as a reference. Both can be classified as envelope-based estimation algorithms.

In accordance with previous studies [42] where envelopes of SCG signals have been used to calculate the HR values, Algorithm 1 consists of applying the Hilbert transform only on two signals: the dorsal-ventral axis of the accelerometer (*z*-axis in our IMU sensor) and laterally from left to right axis of the gyroscope (*y*-axis in our IMU sensor). This axis selection approach is widely adopted in the literature under the hypothesis that the majority of acceleration changes caused by the heart beating are along the *z*-axis (from back to chest) of the accelerometer (see Figure 1) and angular velocity mostly changes along the *y*-axis due to the rotational vibrations of the chest wall induced by the heart beating [22,24].

Assuming y(t) as a generic filtered signal (SCG or GCG), Hilbert transform—as in (2):

(2) $$\hat{y}\left(t\right)=\frac{1}{\pi }\int_{-inf}^{+inf}\frac{y\left(\tau \right)}{t-\tau }d\tau$$

Is applied to obtain the signal’s envelope with a sliding window of 330 ms [42]. These two processed signals represent the retrieved SCG and GCG waveforms.

Algorithm 2 filtering stages are the same as Algorithm 1, but the axes of the accelerometer and the gyroscopes are automatically selected in each trial, making this algorithm more user- and posture-dependent. The power spectral density (PSD) plot identifies the most informative axis per sensor in the frequency domain. Each signal’s PSD was computed using Welch’s overlapped segment averaging estimator. Welch’s technique was used to split the 120 s signal into segments of 30 s, with an overlap value of 50% between segments. Then, the axis exhibiting the highest power spectrum is selected for computing the SCG and GCG waveforms from raw accelerometer and gyroscope data.

After the extraction of SCG and GCG signals with both algorithms, we applied the same filtering pipeline. Considering a generic signal $\hat{y}\left(t\right)$ (SCG or GCG), we first performed a band-pass filter on $\hat{y}\left(t\right)$ with cut-off frequencies of 0.5 Hz and 3 Hz (3rd order filter) to obtain the signal γ(t). The PSD of γ(t) was then computed to identify the dominant frequency ($f_{max}$). Then, the γ(t) signal was rescaled between 0 and 1. All the maxima points were identified on the waveform as all the peaks exceeding 60% of the signal amplitude and separated at least the number of samples equal to $f_{max}$/2. These maxima points identified the AO peaks and are then used to calculate $t_{AO,i}$. AO peaks occurring less than 500 ms after the previous AO peaks were rejected.

**Figure 3:**
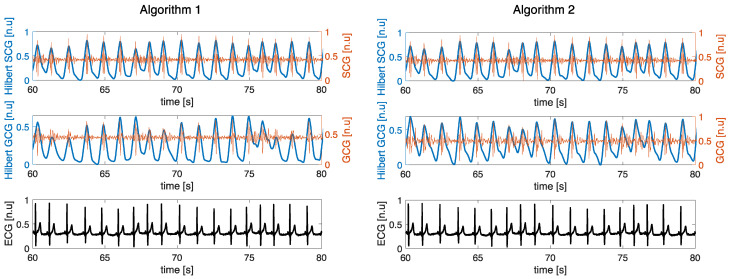
Example of 20 s of SCG and GCG signals (normalized between 0 and 1) obtained with algorithms 1 and 2 after the Hilbert filter application (blue lines) compared to raw SCG and GCG signals (orange lines) and reference ECG (black line, normalized between 0 and 1). In this specific case, Algorithm 2 selected the *z*-axis for SCG, while for GCG the same algorithm selected an axis different from y (default selection for Algorithm 1).

Figure 3 shows the results obtained using Algorithms 1 and 2 to the same raw accelerometer and gyroscope data collected for 20 s from a healthy volunteer. Algorithm 2 selected a more informative axis for the gyroscope since the AO peaks are more distinguishable after applying the Hilbert transform, especially after 75 s.

From AO peaks, the IBI are calculated as differences between timing points of successive AO points ($IBI_{AOAO}$) as in the following equation:

(3) $$IBI_{AOAO,i}=t_{AO}{|}_{n}-t_{AO}{|}_{n-1}$$

where $IBI_{AOAO,i}$ is the *i*-th time interval between consecutive AO peaks in SCG or GCG signals and $t_{AO}{|}_{n}$ denotes the occurrence of *n*-th AO peak in the signal (SCG or GCG).

### 2.2. ECG Signals

Firstly, we resampled the raw ECG signals at a rate of 120 Hz equal to the sampling rate of the skin-interfaced IMU to have the same resolution in the identification of temporal events. The ECG was resampled before windowing the sitting and lying portion of the signals to avoid introducing edge artifacts at each trial. The Pan–Tompkins algorithm was then applied to the recorded ECG waveform to detect the R waves. The time intervals between consecutive R peaks are then calculated as:

(4) $$IBI_{RR,i}=t_{RR}{|}_{n}-t_{RR}{|}_{n-1}$$

where $IBI_{RR,i}$ is the *i*-th time interval between consecutive R peaks in ECG and $t_{RR}{|}_{n}$ denotes the occurrence of *n*-th R peak (see 1st subplot of Figure 2).

### 2.3. Data Analysis

The IBI estimations were performed in a MATLAB environment. To assess the performance of the SCG and GCG signals in estimating IBI values, Bland–Altman analysis was performed. Bland–Altman analysis is one of the most popular methods applied to investigate the agreement between the same measurement extracted with a new measurement technique and an established one [49,50]. Particularly, it was used to obtain the mean of difference (MOD) and the limit of agreement (LOA) values that are typically reported in other studies and extremely useful when comparing our results with the relevant scientific literature [51,52]. This analysis was carried out by considering the two algorithms applied to SCG and GCG data and postures separately to assess the influence of both the algorithms and postures on the IBIs estimations. Additionally, we carried out a correlation analysis on these data to estimate the Pearson correlation coefficient (R).

The IBIs were then used to estimate linear and non-linear HRV indexes. The simplest method of HRV analysis is time domain analysis which is applied to the series of successive IBIs ($IBI_{RR,i}$ and $IBI_{AOAO,i}$) [14].

As linear indexes, in the time domain we calculated:

- Mean heart rate (HR mean);
- Standard deviation of all IBI (SDNN);
- Root mean square of differences (RMSSD);
- The proportion for which the successive IBI differences exceed 50 milliseconds (pNN50);

While in the frequency domain the following:

- the percentage of power of the very low-frequency band (from 0.0033 Hz to 0.04 Hz, VLF);
- the percentage of power of the low-frequency band (from 0.04 Hz to 0.15 Hz, LF);
- the percentage of power of the high-frequency band (from 0.15 Hz to 0.4 Hz, HF)
- the ratio between low and high frequencies (LF/HF).

As non-linear indexes we considered the following measures:

- SD1 as the standard deviation of the orthogonal distances of the $IBI_{i}$/$IBI_{i+1}$ obtained in the Poincaré Plot. SD1 is considered to describe the short-term HRV;
- SD2 as the standard deviation of the orthogonal distances of the $IBI_{i}$/$IBI_{i+1}$ to the length diameter of the ellipse obtained in the Poincaré Plot. SD2 is considered to describe the long-term HRV;
- Ratio between SD1 to SD2 (SD1/SD2) representing balanced ANS;
- Higuchi Fractal Dimension (HFD) measures complexity directly in time series [53];
- Detrended Fluctuation Analysis (DFA), a modified random-walk method, quantifies the fractal-like scaling properties of RR interval series [54];
- Sample entropy (SampEn), a regularity statistic that measures the irregularity (or unpredictability) of the signal [55].

All the HRV parameters were calculated in MATLAB environment from $IBI_{RR}$ and $IBI_{AOAO}$ using the open-source HRVTool [56,57]. Instead of MATLAB, a custom-made algorithm in Java programming language (3EGA, Amsterdam) was used for nonlinear analysis. IBI standardized series were used as input for HFD, DFA, and SampEn. Standardization consisted of extracting the mean interval value for each interval in the time series and dividing the time series by its standard deviation. After the initial calculation, outliers were removed, and means and standard deviations of those series were calculated, as suggested in [12].

To compare all HRV indexes (both linear and nonlinear) that are calculated from SCG and GCG and those extracted from ECG as a reference, Pearson correlation coefficients were calculated per each variable. Then, per each HRV variable, we calculated the mean and standard deviation considering all the values retrieved from the 21 volunteers. Per each HRV index, we applied the one-way ANOVA test considering the ECG extracted value as one group and SCG and GCG ones separately (i.e., EGC vs. SCG, ECG vs. GCG). We considered *p*-values < 0.05 to determine the existence of a statistically significant difference between groups. Moreover, we calculated the percentage error (*E*%) per each HRV index, considering all the values gathered by all the volunteers. Separate analyses were carried out considering SCG and GCG, the posture, and the algorithms as influencing variables.

(5) $$E\%=\frac{HRV-HRV^{reference}}{HRV^{reference}}\cdot 100$$

where *HRV* is a generic HRV index estimated using SCG or GCG, and $HRV^{reference}$ is the HRV index calculated from the reference (i.e., the ECG). In all the cases, we compared 21 values per each considered posture and algorithm.

## 3. Results

**Figure 4:**
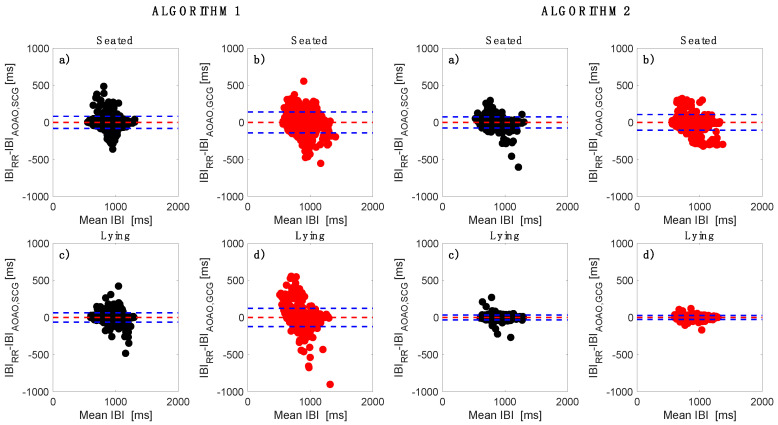
Bland-Altman analysis related to IBI comparison between $IBI_{RR,i}$ and (**a**) $IBI_{AOAO,i}$ estimated from SCG in seated position, (**b**) $IBI_{AOAO,i}$ estimated from GCG in seated position, (**c**) $IBI_{AOAO,i}$ estimated from SCG in lying position and (**d**) $IBI_{AOAO,i}$ estimated from GCG in lying position, for algorithm 1 (panels on the left) and algorithm 2 (panels on the right).

Figure 4 reports the Bland-Altman plots related to the IBI values. The analyses related to data provided by the two algorithms evidenced better performances of Algorithm 2 at parity of posture and raw accelerometer/gyroscope signals. In general, narrower LOAs are achieved when the data collection is performed with the volunteers in lying positions in the case of both SCG and GCG signals. Algorithm 2 applied on gyroscope data collected in a lying position outperforms all the others.

To better clarify the differences between postures and algorithms, Table 1 summarizes the coefficient R obtained from the correlation analysis as well as the MOD and LOAs values of the Bland-Altman analyses. In all the cases, the MOD values were always less than 1.32 ms. Algorithm 2 guaranteed the estimation of more accurate IBI values as the LOAs were narrower and R higher than those of Algorithm 1 in all the conditions and with both SCG and GCG. Between the two postures, the lying position appeared better than the seated one. In the lying position using Algorithm 2, the estimation of IBI with GCG signals is slightly better than the one carried out using SCG signals (comparable MOD values, difference of 7.28 ms in LOAs). All the analyses were carried out on 2464 beats identified in seated positions and on 2274 beats when lying postures were analyzed. In all the cases, R values identified strong linear correlations (the lower value is 0.88) between IBI values retrieved from IMU signals and ECG.

**Figure 5:**
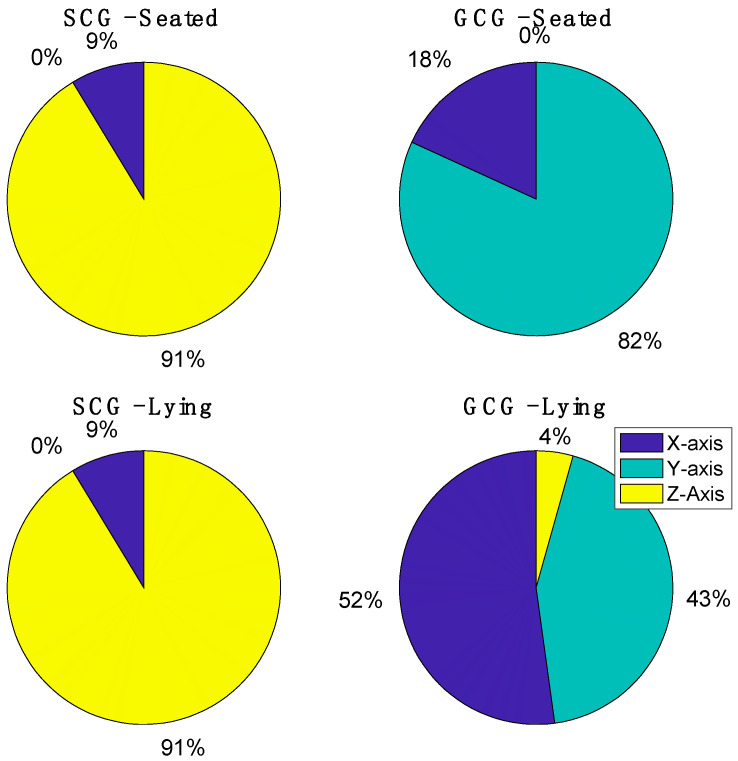
Percentage of selected axes used to retrieve the SCG signals and GCG signals from algorithm 2.

Since Algorithm 2 performed better, we investigated the axis selected from this algorithm to extract the SCG and GCG signal (see Figure 5). From the tri-axial accelerometer, most of the time the algorithm selected the *z*-axis in a seated position (91% of cases) and lying position (91% of cases); differently, on the gyroscope signal the algorithm selected the *x*-axis several times, especially in lying position (52% of cases). This selection justifies the higher performance of GCG in the lying position achieved with Algorithm 2 compared to the one of GCG in the same posture with Algorithm 1 (using only the *y*-axis).

Table 2 and Table 3 reported the mean and standard deviation values of HRV indices calculated from IBI obtained from ECG, SCG, and GCG related to the linear indexes (both time and frequency domains) and non-linear ones, respectively. As Algorithm 2 and lying position evidenced better agreement between IBI estimations, mean, and standard deviation values of calculated HRV indexes are closer to the reference, especially in this condition.

The statical test applied to values of Table 2 and Table 3 demonstrated that the great majority of compared values exhibited very high *p*-values, demonstrating that they are not significantly different in a statistical sense. In the case of Algorithm 2, only a single comparison of series (for LF/HF) showed a significant difference (with *p* < 0.01) in a seated posture, while none in the supine position. For algorithm 1, there were three detected significant differences (for RMSSD, SD1, and SD1/SD2, *p* < 0.01) using GCG in a seated posture.

**Figure 6:**
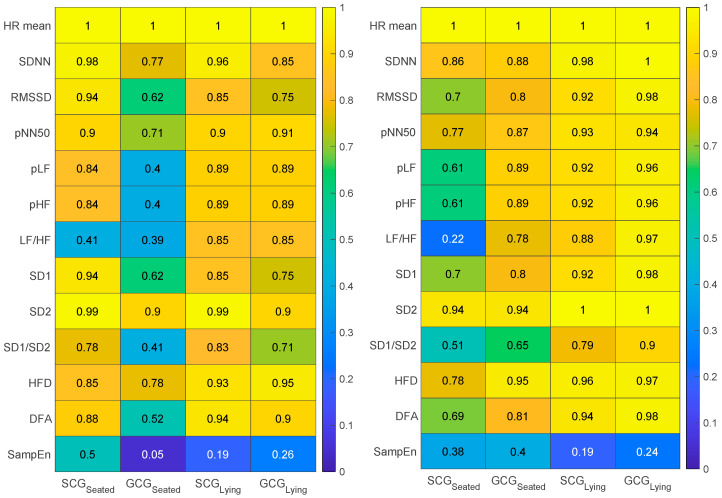
R values (between 0 and 1) obtained in the correlation analysis carried out between values retrieve from SCG and GCG against reference ECG values in both seated and lying position, for both the algorithms.

Figure 6 graphically reports the R values calculated considering the ECG-derived HRV values as a reference. The colors range between 0 (no correlation) and 1 (perfect correlation). In all the cases, we obtained positive R. This analysis evidenced higher R values with volunteers in lying positions. With Algorithm 2, better performance is generally achieved by GCG more than SCG, both in seated and supine posture. Linear HRV indexes estimated from both the SCG and GCG show very strong relations with reference values with the lowest value equal to 0.92 for the SCG (RMSSD). Even the non-linear indexes show a strong correlation, except for the SampEn index (0.19–0.50). Considering all the other indexes, lower values equal to 0.79 and 0.90 for SD1/SD2 index were found for SCG and GCG, respectively.

**Figure 7:**
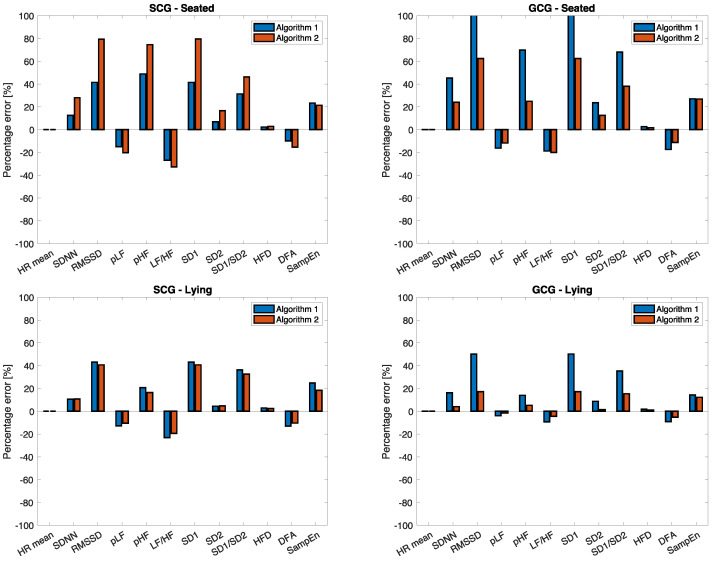
Percentage error between HRV derived from $IBI_{AOAO}$ estimated from SCG and GCG signals and from $IBI_{RR}$ from ECG signal. All the errors are reported in %.

From Figure 7, some indexes show very high percentage error, especially in seated positions with both the SCG and GCG. In lying posture, GCG allows reaching very promising performance with Algorithm 2 with *E*% of 0.01%, 4.04%, 17.19%, −1.56%, 5.19%, −4.50%, 17.20%, 1.39%, 15.30%, 1.10%, −5.25%, 12.12% for HR mean, SDNN, RMSSD, pLF, pHF, LF/HF, SD1, SD2, SD1/SD2, HFD, DFA, and SampEn, respectively. Interestingly, this also shows negligible error when HFD is applied, suggesting its future use. In this posture condition and with GCG, Algorithm 2 allows the error for most HRV indices to be halved.

## 4. Discussion and Conclusions

The aim of this study was to investigate the potential of assessing both linear and non-linear HRV parameters from accelerometer and gyroscope signals (i.e., SCG and GCG). At this scope, an ECG device was used as a reference, and a skin-interfaced IMU sensor to capture accelerations and angular velocities at the xiphoid level. The analysis was carried out at rest under two conditions typical for capturing ECG and HRV variables: seated posture and lying posture (supine). Algorithm 2 (automatically selecting the most informative axes of the accelerometer and the gyroscope in each trial) allows better estimation of IBIs when compared to the ECG reference values. Although the literature indicates some preferential axes of accelerometers and gyroscopes to extract SCG and GCG respectively, the easy-to-implement solution proposed in this paper appears to be better for estimating IBIs. This is beneficial for the subsequent estimation of HRV parameters. Using the GCG signal in the lying posture allows accurate results in the estimation of IBIs in accordance with our previous paper [34] and HRV parameters close to those estimated with ECG.

For all HRV parameters, both the values extracted from SCG and GCG via Algorithm 2 with the subject lying down, we demonstrated the existence of a strong correlation with the values estimated from the ECG signal (minimum value 0.79). The SampEn parameter showed the lowest correlation values and higher percentage error (>15%). Pearson linear correlation coefficients indicate stronger comparability of HRV coefficients calculated on GCG and ECG than on SCG and GCG. These findings are in line with the observations of Yang et al. [45], where GCG was found to be more tolerant to disturbances and inter-subject variability than SCG. The R values are in line with the study of Siecinski et al., with data from a dataset consisting of 29 healthy volunteers but assuming different inter-subject postures [26]. We found that the mean and standard deviation values of HRV indexes are comparable to those found in [33], especially in terms of error between values from ECG and SCG/GCG related to RMSSD, LF/HF, and SD1/SD2, which are the most common indexes used in the research field. Among the non-linear parameters, the estimation of the HFD parameter shows the best performance compared to the values estimated with ECG with errors < 1% and correlation values close to 1. Similar to the DFA parameter with error values of 4%. SDNN and LF/HF values estimated with Algorithm 2 in the supine position exhibited percentage errors less than 4.50% (R equal to 0.99), in line with results obtained using Kubios HRV software on SCG signals [58].

In addition, we found that nonlinear indexes used for further characterization of recorded signals examined other information we might extract from physiological measurements that were not possible to extract via standard time and spectral HRV indexes. HFD confirmed that the level of complexity of examined IBI time series, as expected, demonstrates a healthy level of complexity compared to our prior research (healthy control in several different experiments’ levels of complexity). The complexity of SCG, measured by HFD while sitting is higher than in a supine position. That is also true for the other two signals, GCG and ECG. From our earlier work, the measures of ECG in terms of complexity are within the range of normal healthy ECG variability [12]. All the calculated HFDs have a normal distribution, which is also characteristic of healthy heart dynamics. As stated earlier in Method, artifacts are not removed, so in some cases, higher complexity indexes may result from this (presence of additional components in the signal). DFA, also considered to be a fractal-related measure, confirmed that a great majority of time series exhibit healthy long-range correlations, and a small portion demonstrated that they are not correlated (only one participant in GCG and two in SCG segments within signals), which might be pointing to certain individual differences in dynamics. Sample entropy analysis confirmed that the difference could be seen (as well as in complexity measured by HFD) that there is the difference between the states (i.e., sitting and supine) that is understandable, knowing normal human physiology. However, the added value to this analytic approach is that based on complexity levels (e.g., HFD) and irregularity statistics (e.g., SampEn), we can safely say that SCG and ECG, but also GCG and ECG, exhibit such a high self-similarity that they can interchangeably be used in future research. Interestingly, that is additionally supported by *E*% analyses (where HFD showed minimal error). The difference among signals from the concurrent time series is visible after the second or third decimal place, which is seldom seen in prior measurements. The same applies to entropy-based measurement, hence they demonstrate the same levels of irregularity (that could be interpreted concerning complexity changes, too) so important for healthy human physiology. As a measure of the irregularity (or unpredictability) of a signal, SampEn calculated from ECG is also within the healthy range, similar to those that we could see in healthy controls in our other experiments. Irregularity, or consequentially complexity, is higher when the person is in a supine position. For SCG and GCG, that trend does not hold since in some cases, it is higher in the sitting position (as the GCG in Algorithm 2). Since we are not aware of values reported in the literature, we cannot compare our results with some baseline. SampEn indeed performed poorly on this dataset. However, knowing that it is regularity statistics that we usually apply on the samples of the raw signals like EEG or ECG, the reason for such a performance is probably in the preprocessing steps introduced by Algorithms 1 and 2. To the best of our knowledge, SampEn has shown to be useful in cardiology, but if that signal is reconstructed from mechanical signals, the reconstruction method probably introduced the above-mentioned low performance.

The main limitation of this study is that the sample size is relatively small, and the subjects are all young and healthy. We know so far that complexity measures are changing with healthy aging in a certain way [59] and that electrophysiology-based change is known to be aberrated in different clinical conditions. Nevertheless, that can serve for some future comparison since we already know some benchmarks for healthy dynamics that can be used for further early detection of differences in various disorders in the cardio-pulmonary sense. Collecting GCG and SCG recordings from a larger population would provide better validation of results as more variability will be considered. It should also be noted that the acquisitions are relatively short, so to give more reliability to the data, it could be useful to carry out longer acquisitions and evaluate whether the method allows following any changes in HRV over time. Additionally, further efforts will be devoted to investigating the effect of respiration in HRV analysis. As it has already been demonstrated that a single IMU sensor on the chest is able to capture the respiratory rate [21], in the future we will investigate how the contextual assessment of this vital parameter can help to provide a more accurate assessment of HRV indexes (especially in the frequency domain), which has only been partially demonstrated with studies on a limited number of subjects during ECG monitoring [60,61]. Lastly, the study was conducted only under resting conditions, so motion artifacts that could adversely affect the results were inherently eliminated. Therefore, a possible future work could be to evaluate the performance of this system under more challenging conditions where the subject is in semi-limited motion condition and on patients to see differences in HRV indexes estimated with different technologies.

## Figures and Tables

**Table 1:** IBI analysis: R coefficients, MOD and LOAs values.

	Algorithm 1				Algorithm 2			
	Seated		Lying		Seated		Lying	
	SCG	GCG	SCG	GCG	SCG	GCG	SCG	GCG
**R**	0.95	0.88	0.97	0.90	0.96	0.93	0.99	0.99
**MOD ± LOAs** **[ms]**	0.17 ± 81.66	−1.32 ± 140.48	−0.14 ± 63.68	0.59 ± 122.73	−0.47 ± 75.41	−0.18 ± 105.34	−0.15 ± 33.71	−0.13 ± 26.43
**Number of beats detected**	2464	2464	2274	2274	2464	2464	2274	2274

**Table 2:** Mean and standard deviation for all postures and subjects of HRV indexes related to time (SDNN, RMSSD, PNN50) and frequency (LF, HF, and LF/HF) domain analysis related to algorithms 1 and 2. * means statistically significant (*p* < 0.05).

HRV Index	Alg.	Seated			Lying		
		ECG	SCG	GCG	ECG	SCG	GCG
**HR mean [bpm]**	1	71.33 ± 9.93	71.34 ± 9.91	71.20 ± 9.73	68.29 ± 9.11	68.28 ± 9.11	68.33 ± 9.11
	2		71.30 ± 9.96	71.32 ± 9.93		68.29 ± 9.10	68.29 ± 9.10
**SDNN** **[ms]**	1	67.32 ± 33.66	74.77 ± 36.87	92.06 ± 52.25	65.50 ± 32.42	69.67 ± 30.28	76.52 ± 47.09
	2		80.28 ± 35.44	81.21 ± 50.85		71.02 ± 35.64	66.75 ± 31.77
**RMSSD** **[ms]**	1	55.00 ± 35.26	71.99 ± 43.01	104.48 ± 81.46 *	52.85 ± 34.5	65.31 ± 32.53	76.20 ± 62.46
	2		81.44 ± 46.21	84.31 ± 72.18		69.73 ± 45.47	58.40 ± 34.7
**pNN50** **[%]**	1	30.06 ± 22.76	41.77 ± 25.33	47.83 ± 29.69	26.87 ± 23.36	34.68 ± 22.56	35.49 ± 25.80
	2		46.40 ± 24.14	40.64 ± 23.77		36.77 ± 25.09	32.36 ± 22.94
**pLF** **[%]**	1	56.27 ± 20.80	47.50 ± 18.28	44.51 ± 22.59	55.59 ± 18.91	49.60 ± 19.71	51.26 ± 17.73
	2		43.19 ± 17.85	50.42 ± 22.43		48.36 ± 19.16	54.12 ± 17.89
**pHF** **[%]**	1	43.72 ± 20.80	52.50 ± 18.28	55.482 ± 22.59	44.40 ± 18.91	50.39 ± 19.71	48.73 ± 17.73
	2		56.80 ± 17.85	49.57 ± 22.43		51.63 ± 19.16	45.87 ± 17.89
**LF/HF**	1	2.26 ± 2.78	1.13 ± 0.76	1.16 ± 1.01	1.69 ±1.19	1.31 ± 0.95	1.40 ± 1.11
	2		0.96 ± 0.73 *	1.47 ± 1.23		1.24 ± 0.92	1.54 ± 1.05

**Table 3:** Mean and standard deviation for all postures and subjects of HRV indexes related nonlinear measures for algorithms 1 and 2. * means statistically significant (*p* < 0.05).

HRV Index	Alg.	Seated			Lying		
		ECG	SCG	GCG	ECG	SCG	GCG
**SD1 [s]**	1	0.04 ± 0.02	0.05 ± 0.03	0.07 ± 0.06 *	0.04 ± 0.02	0.04 ± 0.02	0.05 ± 0.04
	2		0.05 ± 0.03	0.05 ± 0.05		0.04 ± 0.03	0.04 ± 0.02
**SD2 [s]**	1	0.09 ± 0.04	0.09 ± 0.04	0.10 ± 0.05	0.08 ± 0.04	0.08 ± 0.04	0.09 ± 0.05
	2		0.09 ± 0.04	0.09 ± 0.05		0.08 ± 0.04	0.08 ± 0.04
**SD1/SD2**	1	0.43 ± 0.13	0.54 ± 0.16	0.68 ± 0.37 *	0.43 ± 0.17	0.55 ± 0.24	0.56 ± 0.24
	2		0.59 ± 0.24	0.58 ± 0.26		0.56 ± 0.23	0.49 ± 0.21
**HFD**	1	1.87 ± 0.09	1.90 ± 0.09	1.91 ± 0.09	1.84 ± 0.10	1.88 ± 0.08	1.87 ± 0.09
	2		1.92 ± 0.09	1.90 ± 0.09		1.89 ± 0.09	1.87 ± 0.10
**DFA**	1	0.83 ± 0.21	0.74 ± 0.21	0.68 ± 0.27	0.90 ± 0.26	0.80 ± 0.25	0.81 ± 0.24
	2		0.69 ± 0.22	0.74 ± 0.25		0.77 ± 0.26	0.84 ± 0.28
**SampEn**	1	1.49 ± 0.34	1.76 ± 0.45	1.77 ± 0.39	1.53 ± 0.28	1.74 ± 0.36	1.71 ± 0.51
	2		1.72 ± 0.38	1.83 ± 0.48		1.83 ± 0.49	1.64 ± 0.29

## Data Availability

The data presented in this study are available on request from the corresponding author. The data are not publicly available due to privacy reasons.
